# Transcriptomic Profiling of Circular RNAs in the Goat Rumen During Fetal and Prepubertal Period

**DOI:** 10.3389/fphys.2022.858991

**Published:** 2022-03-30

**Authors:** Tao Zhong, Cheng Wang, Meng Wang, Siyuan Zhan, Aline Freitas-de-Melo, Linjie Wang, Jiaxue Cao, Dinghui Dai, Jiazhong Guo, Li Li, Hongping Zhang, Lili Niu

**Affiliations:** ^1^Farm Animal Genetic Resources Exploration and Innovation Key Laboratory of Sichuan Province, College of Animal Science and Technology, Sichuan Agricultural University, Chengdu, China; ^2^Departamento de Biociencias Veterinarias, Facultad de Veterinaria, Universidad de la República, Montevideo, Uruguay

**Keywords:** *Capra hircus*, fetus, kids, rumen, circRNA, differentially expression

## Abstract

Circular RNAs (circRNAs) are key regulatory factors with vital functions in various biological activities. However, little has been reported concerning the genetic regulation of circRNAs during rumen development in goats. The aim of this study was to identify the genome-wide expression profiles of circRNAs in the rumen of goats during fetal development and before and after weaning. Histological morphology showed that from the fetal period (days 60 and 135 of gestation) to the prepuberal period (days 60 and 150 of age) the rumen papilla developed gradually, and the thickness of the rumen muscular layer increased. A total of 11,149 circRNAs were identified in the four development stages by RNA-sequencing. From this, 1,518 were differentially expressed circRNAs (DECs). Fifty-eight DECs were up-regulated from 60 to 135 days of gestation, and 93 from day 135 of pregnancy to 30 days after birth. A large proportion (598) of DECs were down-regulated from day 135 of gestation to 30 days after birth. The expression levels of six randomly selected circRNAs were validated by qPCR, and their back-splicing junction (BSJ) sites were also confirmed. Ontology and pathway analyses revealed that the parental genes of DECs were mainly involved in the signaling pathways related to cell proliferation and apoptosis. The interaction network of circRNAs with their target miRNAs showed its involvement in cell proliferation and apoptosis signaling pathways. In conclusion, we identified the genome-wide expression profiles of circRNAs in the rumen of goats during fetal development and before and after weaning. These results provide a basis for further study on the regulatory effect of circRNAs on the development of rumen tissues.

## Introduction

The rumen is the main organ for digestion, absorption, and metabolism in ruminants. The development of the rumen includes anatomic changes (increase in rumen absolute mass and relative size in relation to the other stomach cavities, and growth of the rumen papillae), functional achievement (acquisition of the fermentation capacity and enzyme activity), and microbial colonization (development of the bacteria, fungi, protozoa, and archaea populations) (Wang et al., 2016). From fetal period to adulthood, the morphology and functionality of the rumen epithelium are in continuous development. During the fetal life, rumen function is not required as there is no income from the digestive system. During this period, maternal glucose is the primary energy source for the fetus, being even more critical during the last third of gestation when its greatest growth occurs (Trotta and Swanson, 2021). At the end of gestation, the rumen papillae are entirely developed, and its’ surface is keratinized (Garcia et al., 2012). During the first hours after birth, the ingestion of colostrum is the main energy source for the neonate (Nowak and Poindron, 2006), beginning the income of nutrients through the digestive system. The rumen of the neonate is covered by a smooth, stratified, and squamous epithelium without any prominent papillae, and its physiological functionality is incomplete as the microbiome is not established (Wang et al., 2016). During the first 4 weeks of age, milk is the main energy source for the offspring (Degen and Benjamin, 2003, 2005). Solid food consumption increases gradually with age, being the unique food source for the kids after weaning (Weary et al., 2008). Overall, during ruminant development, the changes in age, diet, and environment are accompanied by rumen growth, and metabolic and physiological adaptations, which are regulated by specific genes.

The studies on rumen development have been mainly focused on changes in its morphology, internal environment, establishment and maintenance of microflora, absorption and transport of nutrients (Lesmeister and Heinrichs, 2004; Jiao et al., 2015; Ragionieri et al., 2016; Wang et al., 2016; Do et al., 2019). However, there is limited information on the candidate genes/ncRNAs related to rumen development. Circular RNAs (circRNAs), a large category of recently known endogenous non-coding RNAs, are characterized as a closed-loop structure covalently linked by the 5′- and 3′- termini (Qu et al., 2015). CircRNAs are generated by the back-splicing of precursor messenger RNAs during the post-transcriptional processes, being more stable than linear RNAs (Jeck et al., 2013). Emerging evidences indicate that circRNAs modulate miRNA activity by competing with endogenous RNA or acting as microRNA sponges, regulating a variety of biological and disease processes (Hansen et al., 2013; Chen et al., 2017; Kristensen et al., 2018; Zhong et al., 2018). Numerous circRNAs have been identified in various tissues of farm animals using the high-throughput sequencing, including yak adipose tissue, goat endometrium, and pig longissimus dorsi muscle (Le Bras, 2019; Song et al., 2019; Wang et al., 2019; Zhang Y. et al., 2020). CircRNAs are ubiquitously expressed and abundant in animals, and they are differentially expressed in diverse types of cells, specific tissues, and developmental stages (Memczak et al., 2013; Ji et al., 2019; Li et al., 2021). Recent studies have identified candidates of miRNAs differently expressed, which are mainly involved in cell proliferation, growth, and apoptosis during the development of goat rumen (Zhong et al., 2017, 2020). Therefore, CircRNAs could participate in those processes, modulating miRNA activity.

Considering all this information, we hypothesized that the universally expressed circRNAs act as potential regulators of the rumen development in goats. The aim of this study was to identify the genome-wide expression profiles of circRNAs in the rumen of goats during fetal development and before and after weaning.

## Materials and Methods

### Ethics Approval and Consent to Participate

The protocol for collecting ruminal biopsies was approved by the Institutional Animal Care and Use Committee of the College of Animal Science and Technology of Sichuan Agricultural University, Sichuan, China, under the permit of No. DKY-2019302081.

### Location, Animals, and General Management

The animals used in this study were raised at the Jianyang Dageda husbandry Co., Ltd., Sichuan, China (34°4′ N; 104°5′ E). Jianzhou Da’er Goat is a meat breed with a large body size, fast growth, high resistance to rough feeding, and desired reproductive performance (China National Commission of Animal Genetic Resources, 2011). All the goats were kept indoors in a naturally illuminated and ventilated shed with playgrounds. Estrous cycles of all the does were synchronized, and after estrus detection, does were inseminated (Day 0). Pregnancy status and fetal number were determined on Day 30 with transrectal ultrasound. Twelve pregnant multiparous goat (2–3 years old), homogenous in body condition score (between 3.0 and 3.5 on a scale of 1–5), were included in this study. The female fetuses were identified by the PCR amplification using the SE47/SE48 sex identification primers (Ennis and Gallagher, 1994). The pregnant goats were fed with 65% forage and 35% concentrate twice daily at 08:00–9:00 h and 17:00–18:00 h. The kids were breast-fed by their mothers and received concentrate supplement *ad libitum*. The kids were weaned at 60 days after birth. The feed nutrition level was designed according to the local feeding standard of Jianzhou Da’er Goat (DB51/T 1750-2014). The weaned goat kids received 70% forage and 30% concentrate twice daily.

### Experimental Groups and Sampling Procedures

The ages for sampling were selected according to the level of rumen development. Considering that the rumen develops gradually during fetal life, and at the end of gestation, the rumen papillae are entirely developed, the fetuses on days 60 and 135 of gestation were selected. After birth, considering that the diet affects rumen development, female kids were selected before and after weaning (30 and 150-days-old) for further study. Rumen tissues during the fetal period were collected at 60 and 135 days of gestation (groups F60 and F135, respectively; *n* = 3 in each group), and after birth, during the prepubertal period, before and after weaning (at 30 and 150 days of age; groups BW30 and AW150, respectively; *n* = 3 in each group). The pregnant does from groups F60 and F135 were anesthetized (intravenous injection of Zoletil 50^®^, 4 mg/kg, Carros, France) according to Zhong et al. (2017), followed by the cesarean section to separate fetus. The rumen of fetuses was sampled by a veterinary surgery immediately after a cesarean. The kids from groups BW30 and AW150 were euthanized with anesthesia followed by immediate bleeding. Thereafter, the rumen tissues were dissected, and each sample was divided into two similar pieces. One piece was immediately frozen in liquid nitrogen and then stored at −80^°^C for the following RNA-Seq analysis. The other half of the rumen sample was rinsed in phosphate buffer saline (PBS) solution and fixed with 4% formaldehyde for hematoxylin and eosin staining.

### Hematoxylin and Eosin Staining

The fixed rumen samples were dehydrated in increasingly concentrated ethanol (80°, 90°, and 95°), dehydrated in xylene, and embedded in paraffin wax. Sections (5-μm) were cut using a microtome (LeicaRM2016, Leica Microsystems, Wetzlar, Germany) perpendicular to the longitudinal axis of the rumen and placed on glass slides. The hematoxylin-eosin stained sections were captured using an Olympus BX51 microscope (Olympus, Tokyo, Japan) equipped with a Nikon DS-Fi1 camera (Nikon, Tokyo, Japan).

### Rumen RNA Isolation, Library Preparation, and Sequencing

Total RNA was extracted from rumen tissues using the TRIzol reagent (Invitrogen, CA, United States). The quality, quantity, and integrity of RNA were detected by the NanoDrop spectrophotometer (Thermo Fisher Scientific, DE, United States) and Agilent 2100 Bioanalyzer (California, United States). Approximately 5 μg of total RNA was used to remove ribosomal RNAs by the Epicenter Ribo-zero™ rRNA Removal Kit (Epicenter, Madison, WI, United States) and then randomly interrupt rRNA-depleted RNA by adding the Fragmentation Buffer. Sequencing libraries were generated with the NEB Next^®^ Ultra™ Directional RNA Library Prep Kit (New England Biolabs, Beverly, MA, United States). Lastly, the libraries were qualified with the Agilent 2100 Bioanalyzer. The clustering of the index-coded samples was performed on a cBot Cluster Generation System using the HiSeq PE Cluster Kit v4 cBot (Illumina, San Diego, CA, United States). After cluster generation, the library preparations were sequenced on the Illumina HiSeq 4000 platform, and 150 bp paired-end reads were generated.

### Sequencing Data Analysis, circRNAs’ Identification, and Expression

Raw reads were filtered to acquire clean reads by removing adapter-containing reads, poly-N-containing reads (over 5%), and low-quality reads using the FASTQC (Cock et al., 2010). The clean reads were aligned to the goat reference genome (ARS1, GCF_001704415.1) using the TopHat v2.0.9. To predict circRNAs, the sequence alignment map format file was generated by aligning to the ARS1 using the BWA-MEM algorithm (Houtgast et al., 2018), which was performed in the CIRI software (Gao et al., 2015). Prediction detected reverse concatenated reads (at least two unique back-spliced reads whereas obey the GU AG rule) using default parameters.

The relative expression of each circRNA was estimated using the mapped back-splicing junction (BSJ) reads and normalized by transcripts per million reads (TPM) (Arcinas et al., 2019). The DESeq2 package was applied to identify differentially expressed circRNAs (DECs) in each comparison between two different developmental stages. The *p*-value was adjusted by the Benjamini-Hochberg approach for controlling the false discovery rate (FDR < 0.05). The circRNAs with adjusted FDR < 0.05 and | log_2_(Fold Change)| > 1 were considered as DECs.

### GO Enrichment, KEGG Pathway, and CircRNA-miRNA Network

The GO and KEGG analyses were performed using the DAVID tool (Huang et al., 2009), and KOBAS software (Xie et al., 2011) was used to explore the functions of the parental genes of the DECs identified in the rumen tissues. The corrected *p*-value < 0.05 was used as the threshold to define the significant enriched GO terms or KEGG pathways.

CircRNAs are proposed to regulate gene expression through competing endogenous RNAs acting as miRNA sponges (Hansen et al., 2013). The target miRNAs, which could be bound to the circRNAs, were predicted using the miRnada and regRNA 2.0 database (Chang et al., 2013) with a set of default parameters. Then, the goat rumen miRNAs from our previous studies (Zhong et al., 2017), which were identical to the above predicted miRNAs, were used to construct the circRNA-miRNA network by the Cytoscape software (Shannon et al., 2003).

### Validation of CircRNAs by qRT-PCR, Sequencing, and RNase R Resistance

The circRNAs sequencing data was validated by randomly selecting six DECs to assess the reliability of the obtained results. Firstly, divergent primers (Supplementary Table 1) were designed and used to detect the expression by the RT-qPCR, and the BSJ sites of each circRNA were confirmed by Sanger sequencing. The expression of the selected circRNA was determined by the 2^–ΔΔCt^ method with the internal *GAPDH* gene. Amplified fragments of PCR were visualized using 2% agarose gel electrophoresis and then performed cloning sequencing on an ABI 3730XL DNA analyzer (Applied Biosystems, CA, United States). Secondly, the resistance of selected circRNAs to exonuclease digestion was assessed by RNase R assay. Total RNA was treated with the RNase R reagent according to the manufacturer’s instructions (Geneseed, Guangzhou, China). cDNA was synthesized using the random primer and OligdT primer, respectively. Then, PCR was performed to compare the exonuclease resistance of circular and linear transcripts. Data were displayed as mean ± SEM and were considered statistically significant when the *P*-value was less than 0.05.

## Results

### Histological Observation of the Goat Rumens

**FIGURE 1:**
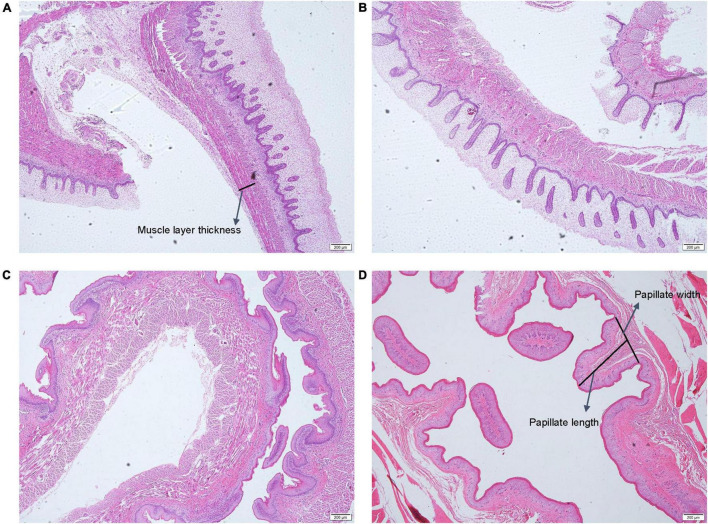
General view of goat rumen tissue during fetal and prepubertal periods. Photomicrograph of rumen wall of the fetal goat at F60 **(A)** and at F135 **(B)** days of gestation, and at 30 (BW30) and 150 days (AW150) of age **(C,D)**, respectively. Bar, 200 μm.

Evident stratification of the rumen wall was observed in the rumen tissues from the F60 group, and the rumen papilla was present (Figure 1A). The papilla of the rumen became longer, and the layers of the rumen wall were thicker in the F135 group than those in theF60 group (Figure 1B). The length and width of the rumen papilla were longer and wider, and the thickness of the muscular layer was wider in the BW60 than in the AW150 group (Figures 1C,D).

### Expression Profiles of CircRNAs in Goat Rumens

**FIGURE 2:**
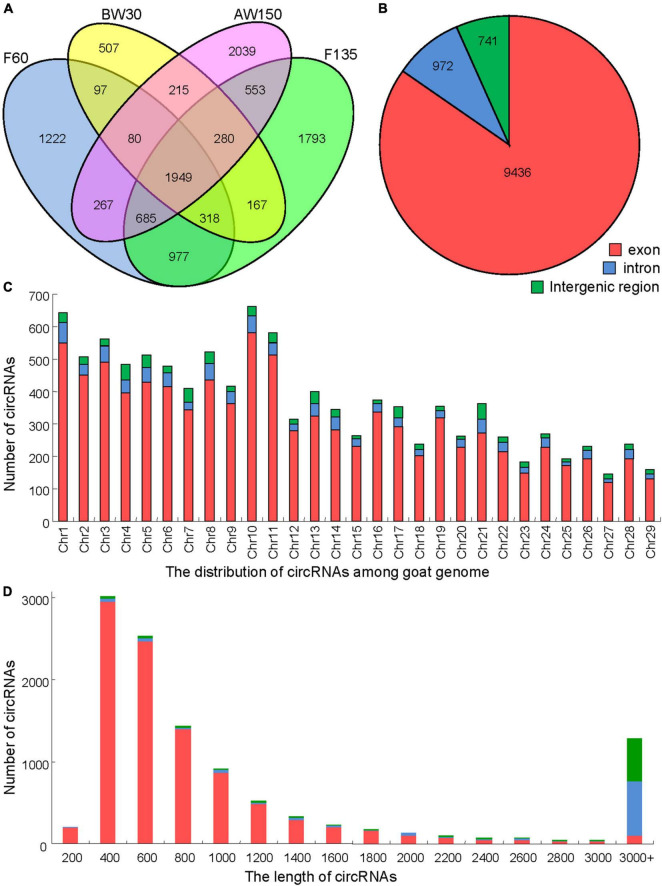
Properties of circRNAs in goat rumen tissue samples. **(A)** Venn diagram showing the number of circRNAs either shared between or uniquely expressed in goat rumen tissues during fetal and prepubertal periods. **(B)** Pie chart showing the prevalence of different circRNA types. circRNAs from introns are represented by the orange section, while the blue region indicates circRNAs from exons. **(C)** Distribution of circRNAs on the chromosomes of the goat reference genome. **(D)** Lengths of the circRNAs.

A total of 1,492,819,832 clean reads were obtained from the rumen tissues collected from the groups of F60, F135, BW30, and AW150, to capture the expression landscape of circRNAs during rumen development. Each library generated more than 11 GB clean bases after removing the adapter sequences and low-quality reads (Table 1). Over 91% of the valid reads were successfully mapped to the goat reference genome ARS1. A total of 11,149 circRNAs were identified, some of them are listed (Supplementary Table 2), including 8,388 circRNAs expressed in the fetal rumens (F60 and F135), 7,157 circRNAs in the prepubertal rumens (BW30 and AW150), and 1,949 common circRNAs across the four developmental stages (Figure 2A). These circRNAs identified in the rumen tissues were mainly derived from exons (up to 84.6%, Figure 2B) and displayed a broad distribution on each chromosome with the highest number on chromosome 10 (Chr10, 663) but the lowest number on Chr27 (145, Figure 2C). We further analyzed the length distribution of the circRNAs, ranging from 400 to 1,400 nt among 8,966 circRNAs (80.42%, Figure 2D).

**TABLE 1:** Summary of circRNAs sequencing results in goat rumen tissues at four developmental stages.

Stage	Sample	Clean reads	Mapped reads	Clean bases (G)	N (%)	Q20 (%)	Q30 (%)	GC content (%)
F60	L01	101,351,038	101,308,832 (99.96%)	12.76	0.00	96.47	91.56	54.17
	L02	94,353,938	94,313,508 (99.96%)	11.88	0.00	96.26	91.12	51.40
	L03	94,119,806	94,088,014 (99.97%)	11.85	0.00	96.85	92.02	54.71
F135	L04	111,335,878	111,311,328 (99.98%)	14.02	0.00	96.70	91.69	56.61
	L05	128,167,378	128,143,752 (99.98%)	16.14	0.00	96.86	92.05	54.41
	L06	111,206,848	111,172,694 (99.97%)	14.00	0.00	96.64	91.59	54.52
BW30	L07	136,101,318	135,440,790 (99.51%)	20.36	0.01	96.08	91.25	48.11
	L08	179,449,110	178,770,854 (99.62%)	26.86	0.01	95.99	91.12	47.24
	L09	141,793,140	141,528,782 (99.81%)	21.22	0.01	95.96	91.07	46.99
AW150	L10	122,153,380	120,952,694 (99.02%)	18.25	0.01	96.05	91.16	49.72
	L11	116,057,964	115,143,978 (99.21%)	17.33	0.01	95.55	90.47	48.14
	L12	156,730,034	143,363,796 (91.47%)	23.46	0.01	96.47	91.41	52.66

### Differentially Expressed CircRNAs in Goat Rumens

**FIGURE 3:**
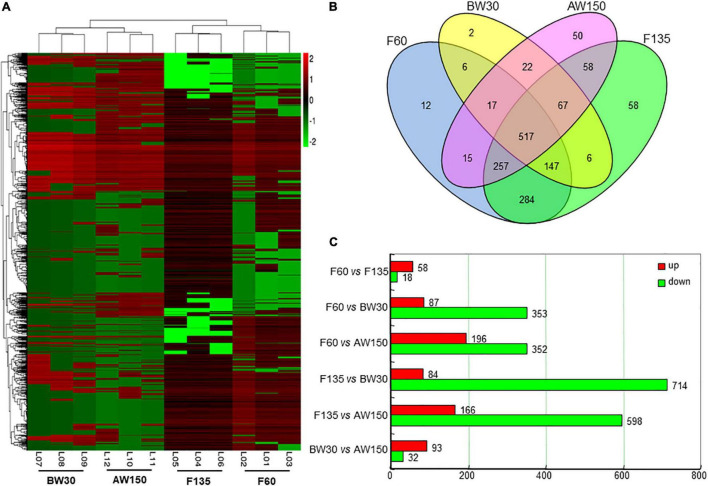
**(A)** Hierarchical cluster analysis (heat map) represents all differentially expressed circRNAs (DECs) between F60 vs. F135, BW30 vs. AW150 groups. Red and green denoted high and low expressions, respectively. **(B)** Venn diagram showing the DECs at the four developmental stages. **(C)** Numbers of up-regulated or down-regulated circRNAs in goat rumens during fetal and prepubertal periods.

A total of 1,518 circRNAs were found to be differentially expressed in the rumen tissues between the four development stages. 76, 798, and 125 DECs were identified between the comparisons of F60 and F135, F135 and BW30, BW30 and AW150 groups, respectively (Supplementary Table 3), and their expression profiles are shown using the hierarchical cluster (Figure 3A). The result showed that the expression of DECs was clearly distinguished and clustered into the fetal (F60 and F135) and prepubertal periods (BW30 and AW150), represented by a well-consistent pattern among the three samples at each developmental stage. A Venn diagram was constructed to explore the interactions between the DECs in each comparison, represented by 1,518 rumen DECs (Figure 3B). It is noticed that 12, 58, 2, and 50 DECs were the stage-specific DECs in the relative period, and 517 DECs were jointly identified in the four developmental stages. Totally, 125 circRNAs were significantly altered in the AW150 rumens compared with the BW30 rumens. Of which, 93 circRNAs were significantly up-regulated, and 32 circRNAs were remarkably down-regulated in AW150 rumens (Figure 3C). Notably, we observed more numbers of up-regulated DECs in the comparisons between F60 vs. F135, BW30 vs. AW150 groups, whereas a large number of down-regulated DECs were identified in the comparison between F135 and BW30 groups (Figure 3C). In addition, we selected six circRNAs and compared them with human circRNAs according to their parental genes and sequences, and found that these six circRNAs had high sequence similarity with human circRNAs (Supplementary Figure 1).

### Biological Functions of the Parental Genes of Differentially Expressed CircRNAs

**FIGURE 4:**
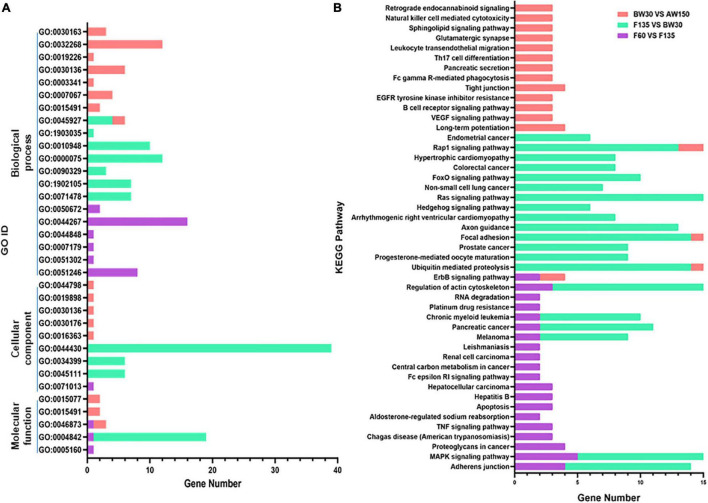
**(A)** The significantly enriched GO terms (*p* < 0.01) in the biological process, molecular function, and cellular component categories. **(B)** Top 20 KEGG enrichment pathways.

GO term and KEGG pathway analyses were performed to explore the function of the parental genes of the DECs identified in the three comparisons between the following groups: F60 vs. F135, F135 vs. BW30, and BW30 vs. AW150. 80, 158, 144 GO terms were identified in these three comparisons (Supplementary Table 4). The biological progress was the largest component of the GO terms (Figure 4A). When comparing F60 with F135 groups, and BW30 with AW150 groups, the parental genes of DECs were involved in cellular protein metabolic process (GO:0044267, *p* < 0.01), regulation of cell division (GO:0051246, *p* < 0.01), and regulation of cell division (GO:0051302, *p* < 0.01). When comparing F135 with BW30 groups, the most enriched pathway was the cytoskeletal part (GO:0044430, *p* < 0.01).

KEGG pathway analysis can reveal molecular interactions and the pathways associated with genes. The MAPK signaling pathway related to cell proliferation and apoptosis was identified across the three comparisons (Figure 4B). In contrast, the Ras and Rap1 signaling pathways were enriched in F135 compared with BW30, and in BW30 relative to AW150 (Figure 4B). In addition, the Adherens junction was enriched in F60 compared with F135, and in F135 vs. BW30, with the highest enrich factor in this latter comparison. Enrichment information of all the KEGG pathways in three comparisons is shown in the Supplementary Table 5. Subsequently, the pathways of the stage-specific DECs in either F135 or AW150 were represented in the Supplementary Figure 1. Their parental genes were also significantly enriched in the MAPK, Rap1, and Ras signaling pathways in F135 and AW150 groups. Furthermore, we selected several circRNAs with high junction ratio and performed KEGG analysis on their parental genes. Interestingly, we found that some of them were involved in RNA transport and basic transcription factors (Supplementary Table 6).

### Construction of the CircRNA-miRNA Network

**FIGURE 5:**
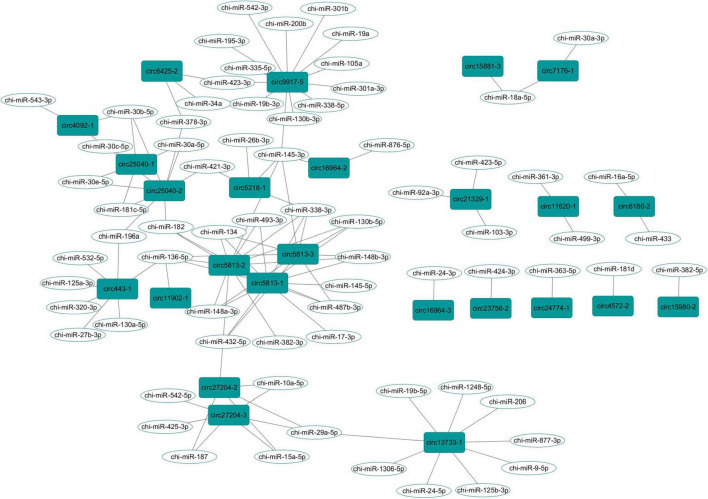
The circRNA-miRNA interaction network in the goat rumen during fetal and prepubertal periods, Including 27 circRNAs and 71 miRNAs.

In order to explore the role of the parental genes of circRNAs involved in the MAPK signaling pathway, we used RegRNA2.0 to predict binding miRNAs with a score of 155 and minimum free energy of 15 kcal/mol as the criteria. Twenty-five circRNAs were found to be combined with 71 binding miRNAs (Figure 5 and Supplementary Table 7). Among them, 19 circRNAs had multiple miRNA targets, and the circRNAs derived from *MAP3K5* (circ9917-5) or *DUSP16* (circ5813-1, circ5813-2, and circ5813-3) had the most number of target miRNAs.

### Verification of the Obtained CircRNAs

**FIGURE 6:**
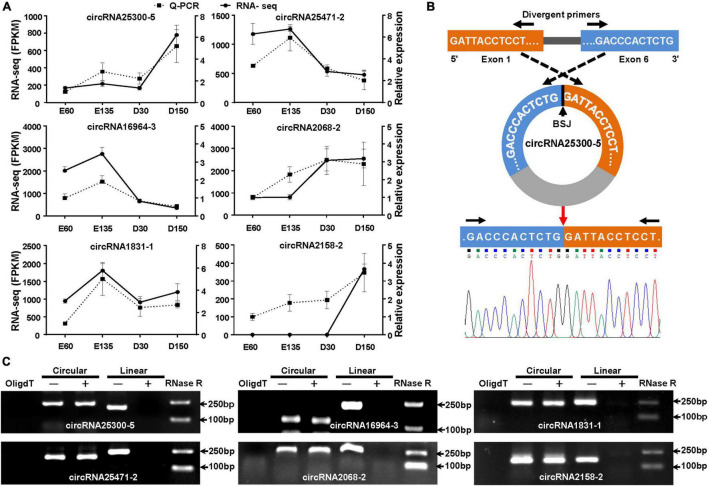
Validation of DECs in goat rumens in the fetal and prepubertal periods. **(A)** Expression profiles of the six randomly selected circRNAs based on RNA-Seq and qPCR. **(B)** Schematic for back-splicing junction (BSJ) validation of circRNAs, represented by circ25300-5. The (BSJ) sequence was confirmed by Sanger sequencing. **(C)** RNase R assay for the circularity of the selected circRNAs by resistance to exonuclease digestion. OligdT shows the template cDNA for RT-PCR synthesized with the OligdT primer, while others were generated using random primers. +, indicates the samples were treated with exonuclease.

Six DECs (circRNA25300-5, circRNA25471-2, circRNA16964-3, circRNA2068-2, circRNA1831-1, and circRNA2158-2) were randomly selected to validate the reliability of RNA-Seq data. the qPCR results of all the six circRNAs showed transcription tendencies highly similar to those in the RNA-Seq data (Figure 6A). The BSJ sequences of the six circRNAs were also verified by Sanger sequencing using divergent primers, represented by circRNA25300-5 (Figure 6B). Subsequently, we performed RT-PCR to compare the exonuclease resistance of circular and linear transcripts. All the six circRNAs showed a resistance to the RNase R digestion (Figure 6C). In addition, the PCR reaction, using the cDNA synthesized by OligdT primer, did not amplify any band because the circRNAs lacked 3′ poly-A tail. These verifications indicated that the circRNAs sequencing data were reliable.

## Discussion

To the best of our knowledge, this is the first study that identified the genome-wide expression profiles of rumen circRNAs during fetal development and before and after weaning in ruminants. The development of the rumen changes with age and is determined by genetic regulation (Zhong et al., 2020), diet composition (Xue et al., 2019), and microbial colonization (Lin et al., 2019). In the early stage of rumen development at the second week of newborn kids, the volatile fatty acids generated by the microbiota can interact with the host transcriptome and mRNA to influence the metabolism of rumen tissue and epithelial development (Lin et al., 2019). In terms of transcriptome analysis, the involvement of miRNA and mRNA in the regulation of rumen development has been reported (Zhong et al., 2017, 2020; Do et al., 2019), but the regulatory mechanism of circRNAs in rumen development has rarely been reported (Do et al., 2019). In the present study, we identified a total of 11,149 circRNAs during the fetal (at 60 and 135 days of gestation) and prepubertal (at 30 and 150 days of age) periods in goats, among which 1,518 circRNAs were differentially expressed. These results suggest that these circRNAs may play an active role in rumen development. We found that most of the DECs were up-regulated during the development of the rumen in the fetal period (F60 and F135), and in the pre- and post-weaning period (BW30 and AW150). In fetal and prepubertal periods, the number of down-regulated circRNAs in the rumen tissues was more than that of up-regulated circRNAs. We proposed that those circRNAs may be involved in the adaptation of rumen function, together with the changes in age and diet.

Previous studies have shown that rumen development is related to specific biological pathways, including pteridine-containing compounds, calcium signaling, and transcription factor PPAR-α. In this study, we found that the parental genes of DECs were significantly enriched in the signal pathways related to cell proliferation and apoptosis, such as MAPK (Han et al., 2020), Ras (Takino et al., 2019), FoxO (Xie et al., 2019), and Rap1 signaling pathways (Zhou et al., 2020). In addition, 83 circRNAs were only expressed in AW150 when we compared BW30 vs. AW150 groups (Supplementary Table 3), suggesting that the functions of circRNAs change with weaning and/or with age. The parental genes in the rumen tissue from the AW150 group were also significantly enriched in MAPK, RAS, and RAP1 signaling pathways (Supplementary Figure 2), indicating that such changes were related to rumen development. In our previous study, the target genes of differently expressed miRNAs identified in goat rumens were mainly enriched with similar signal pathways, such as MAPK, Ras, and Jak-STAT signaling pathways (Zhong et al., 2020). In view of the KEGG analysis, we hypothesized that these related pathways might play an essential role in the development of rumen epithelial cells.

CircRNAs could act as the sponges of miRNAs by competitively binding to miRNAs and modulating the activities of their target genes (Li et al., 2018). In fact, one circ9917-5, derived from the *MAP3K5* gene, had 13 miRNA targets (Figure 5). We proposed that this circRNA could play its function as a miRNA sponge. In gastric cancer cells, circDUSP16 acted as a sponge of miR-145-5p and affected cell proliferation (Zhang Z. et al., 2020). Interestingly, three circRNAs (circ5813-1, circ5813-2, and circ5813-3) were derived by cyclization of the *DUSP16* transcripts, which could bind with chi-miR-145-5p in this study. Thus, we suggest that these three circRNAs may play biological roles in the proliferation of rumen epithelial cells. In addition, circ5813-1, circ5813-2, and circ5813-3 were found to have a binding site of miR-148a-3p (Supplementary Table 6). In the previous study, we found that chi-miR-148a-3p could regulate the proliferation of gastric mucosal epithelial cells (Zhong et al., 2020). Therefore, it was speculated that these circRNAs could act as molecular sponges of miR-148a-3p in the rumen tissues. Several miRNAs have been identified as the key regulators of early rumen development in cattle, including miR-29b, miR-145, and miR-493 (Do et al., 2019). In the present study, we found that chi-miR-29a-5p, chi-miR-145-5p, chi-miR-145-3p, and chi-miR-493-3p could bind to more than one circRNAs (Supplementary Table 6). We speculated that those circRNAs could bind with miRNAs during rumen development and play potential regulatory roles during rumen development in goats.

The molecular changes observed in the rumen tissue from goats during fetal and prepubertal periods were accompanied by morphological modifications. In this study, it was observed that the rumen papilla was already present in the fetal period. During the prepubertal period, the rumen papilla and the thickness of the muscle layer increased rapidly, probably due to the stimulation of the diet and the increase in age. In this sense, the rumen papilla can increase the surface area of the rumen epithelium, thereby prompting the absorption of short-chain fatty acids and minerals in the rumen (Lavker and Matoltsy, 1970). Previous studies have found that volatile fatty acids in the diet can enhance rumen development (Gorka et al., 2011). These results, as a whole, indicate that circRNAs could regulate the rumen development in goats.

In summary, a total of 11,149 circRNAs and 1,518 DECs were found in four developmental stages of goat rumens. Among them, the number of DECs between F135 and BW30 was the largest, suggesting that rumen development before and after birth was regulated by circRNA. GO and KEGG analyses showed that the parental genes of circRNAs were involved in cell division, proliferation, and apoptosis. Meanwhile, we predicted some of the circRNA target miRNAs and constructed a circRNA-miRNA network to expand our understanding. Circ5813-1 was predicted to combine with 12 binding miRNAs, so it could be a key circRNA involved in the process of rumen development in goats. Overall, our results can support further studies on the involvement of circRNA in the regulatory mechanisms of rumen development of goats.

## Data Availability Statement

The datasets presented in this study can be found in online repositories. The names of the repository/repositories and accession number(s) can be found below: https://www.ncbi.nlm. nih.gov/bioproject/, PRJNA720177.

## Ethics Statement

The animal study was reviewed and approved by the Institutional Animal Care and Use Committee of the College of Animal Science and Technology of Sichuan Agricultural University.

## Author Contributions

TZ and LN conceived, designed the experiments, and contributed to reagents and materials. CW and MW performed the experiments. CW, SZ, and JC analyzed the data. LW, DD, JG, LL, and HZ participated in sample collection. CW and TZ wrote the manuscript. AF-M contributed to the interpretation and made substantial contributions to the manuscript. All authors have read and approved the final manuscript.

## Conflict of Interest

The authors declare that the research was conducted in the absence of any commercial or financial relationships that could be construed as a potential conflict of interest.

## Publisher’s Note

All claims expressed in this article are solely those of the authors and do not necessarily represent those of their affiliated organizations, or those of the publisher, the editors and the reviewers. Any product that may be evaluated in this article, or claim that may be made by its manufacturer, is not guaranteed or endorsed by the publisher.

## Supplementary Material

Supplementary Figure 1 Sequence alignment of goat circRNA and human circRNA.

Supplementary Figure 2 Pathway analysis of the parental genes of differentially expressed circRNAs (DECs). **(A)** The top 20 pathways of the parental genes of the stage-specific DECs in F135. **(B)** The top 20 pathways of the parental genes of the stage-specific DECs in AW150.
