# Supplementary figures and images for: Transcriptomic Profiling of Circular RNAs in the Goat Rumen During Fetal and Prepubertal Period
Source: Front Physiol. 2022 Mar 30;13:858991. doi: 10.3389/fphys.2022.858991 (PMC9006873; doi:10.3389/fphys.2022.858991)

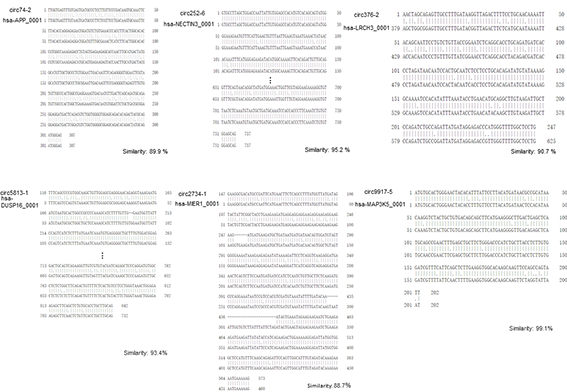

Supplement: Supplementary Figure 1 — Sequence alignment of goat circRNA and human circRNA. [file Image_1.TIF]

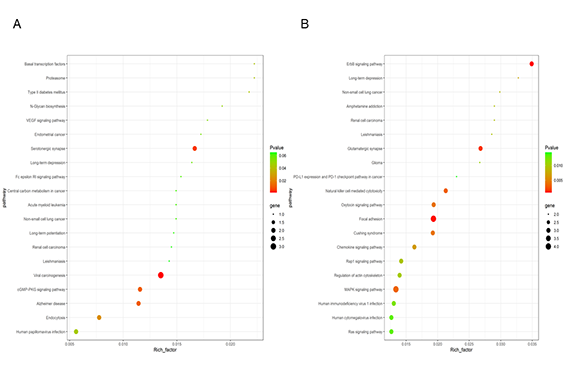

Supplement: Supplementary Figure 2 — Pathway analysis of the parental genes of differentially expressed circRNAs (DECs). (A) The top 20 pathways of the parental genes of the stage-specific DECs in F135. (B) The top 20 pathways of the parental genes of the stage-specific DECs in AW150. [file Image_2.TIF]
