# Supplementary material for: Transcriptomic Profiling of Circular RNAs in the Goat Rumen During Fetal and Prepubertal Period
Source: Front Physiol. 2022 Mar 30;13:858991. doi: 10.3389/fphys.2022.858991 (PMC9006873; doi:10.3389/fphys.2022.858991)
Supplement: Supplementary file 3 [file Table_1.DOC]

**Table S1. The primers used in this study.**

| Gene | Primer | Primer Sequence (5'-3') | Size | Tm °C |
| --- | --- | --- | --- | --- |
| *Expression determined by qPCR* | | | | |
| circRNA25300-5 | Forward | GAGGCATTTGTCATCAGCGT | 77 | 56.6 |
|  | Reverse | TGTTTCAATGATATCTGCCCCAG |  |  |
| circRNA25471-2 | Forward | TTCTGTACTGAGGGTTCGCA | 166 | 59.4 |
|  | Reverse | CTTCTGGGAGCGGATTGAAC |  |  |
| circRNA16964-3 | Forward | TCCTTATCCTGTGGGCAAAC | 91 | 59.4 |
|  | Reverse | CATTCACAGCCACGCCTAAA |  |  |
| circRNA2068-2 | Forward | GATGACATTTCCAGCCCAGG | 218 | 61.3 |
|  | Reverse | GTCACTGGAGCTGGCAAATC |  |  |
| circRNA1831-1 | Forward | CTCCTGTGCTCGTTCCAACT | 100 | 59.4 |
|  | Reverse | GCGATTGGTTCTATCTCTTTCCC |  |  |
| circRNA2158-2 | Forward | CATGATCATTGCGCTGCTTG | 189 | 60.5 |
|  | Reverse | CCCTCTTTCACATGGACCCT |  |  |
| GAPDH | Forward | GCAAGTTCCACGGCACAG | 249 | 61.3 |
|  | Reverse | GGTTCACGCCCATCACAA |  |  |
| *Linear mRNA amplification using convergent primers* | | | | |
| circRNA25300-5 | Forward | CCCATCTGTGGAGAGACCAG | 207 | 58.4 |
|  | Reverse | GAAATAAAGACAGGCCGGGGA |  |  |
| circRNA25471-2 | Forward | TGGCTTTTGGGAGTTCCGTG | 246 | 58.4 |
|  | Reverse | AATGGATAGCTGCTGTGGCTT |  |  |
| circRNA16964-3 | Forward | CGTTCTGCAGGAAGCTTTAGG | 217 | 58.4 |
|  | Reverse | GCAAAGGTGGTTTGCCCACA |  |  |
| circRNA2068-2 | Forward | CTCCAGTGACTCCACAACCTC | 252 | 58.4 |
|  | Reverse | TTGTCACAACTCCAACGAGA |  |  |
| circRNA1831-1 | Forward | CGCACTGGTGTTGAATCCTG | 248 | 58.4 |
|  | Reverse | AGTAGGCTGTGTGACCTCCA |  |  |
| circRNA2158-2 | Forward | TGACTCGGATCCGGACCTTT | 159 | 57.0 |
|  | Reverse | CGACTGTAAACTCGTGCGTTC |  |  |
| *BSJ identification using divergent primers* | | | | |
| circRNA25300-5 | Forward | GAGGCATTTGTCATCAGCGT | 241 | 55.0 |
|  | Reverse | TTAGTTGGACCCAGAGCTCC |  |  |
| circRNA25471-2 | Forward | CATCCAATGACTGTGAGGGG | 194 | 59.0 |
|  | Reverse | CTGTCCTTGAACTTCCACGG |  |  |
| circRNA16964-3 | Forward | CAGAAAGGGGAAAGACAAAC | 128 | 57.0 |
|  | Reverse | ATTCACAGCCACGCCTAAAG |  |  |
| circRNA2068-2 | Forward | CCAGTTGTGCCAGCTCATAA | 223 | 55.0 |
|  | Reverse | TAACCCCTGGCTTTGGAGAC |  |  |
| circRNA1831-1 | Forward | CAGTAACACCTAAGCCTC | 233 | 58.0 |
|  | Reverse | ATTCAACACCAGTGCGAT |  |  |
| circRNA2158-2 | Forward | GCTTTGCTGTGCTGGATTTC | 153 | 56.0 |
|  | Reverse | CTTTCACATGGACCCTTGGC |  |  |
