# Supplementary material for: Transcriptomic Profiling of Circular RNAs in the Goat Rumen During Fetal and Prepubertal Period
Source: Front Physiol. 2022 Mar 30;13:858991. doi: 10.3389/fphys.2022.858991 (PMC9006873; doi:10.3389/fphys.2022.858991)
Supplement: Supplementary file 8 [file Table_6.DOCX]

| KEGG pathway | Count | Genes | Fold Enrichment | Bonferroni | Benjamini | FDR |
| --- | --- | --- | --- | --- | --- | --- |
| Axon guidance | 1 | SEMA3F | 11.91736 | 1 | 1 | 1 |
| Spliceosome | 1 | THOC2 | 11.00763 | 1 | 1 | 1 |
| Fanconi anemia pathway | 1 | FANCL | 28.27451 | 1 | 1 | 1 |
| Ubiquitin mediated proteolysis | 1 | FANCL | 10.60294 | 1 | 1 | 1 |
| Retrograde endocannabinoid signaling | 1 | RIMS1 | 14.27723 | 1 | 1 | 1 |
| Viral carcinogenesis | 1 | GTF2A1L | 7.357143 | 1 | 1 | 1 |
| Synaptic vesicle cycle | 1 | RIMS1 | 22.53125 | 1 | 1 | 1 |
| RNA transport | 1 | THOC2 | 8.792683 | 1 | 1 | 1 |
| Basal transcription factors | 1 | GTF2A1L | 29.42857 | 1 | 1 | 1 |

**Table S6:** **The KEGG pathway analysis of the parental genes of circRNAs with high junction ratio**
